# Thermal Properties of TiO_2_NP/CNT/LDPE Hybrid Nanocomposite Films

**DOI:** 10.3390/polym10111270

**Published:** 2018-11-15

**Authors:** Moustafa M. Zagho, Mariam Al Ali AlMaadeed, Khaliq Majeed

**Affiliations:** 1 Materials Science and Technology Program, College of Arts and Sciences, Qatar University, Doha 2713, Qatar; 2 Center for Advanced Materials, Qatar University, Doha 2713, Qatar; khaliqmajeed@gmail.com; 3 Department of Chemical Engineering, COMSATS University Islamabad, Lahore Campus, Lahore 54000, Pakistan

**Keywords:** polymer composites, LDPE, TONPs, CNTs, morphological properties, thermal properties

## Abstract

This work aims to investigate the effect of hybrid filler concentration on the thermal stability of low-density polyethylene (LDPE) matrices. LDPE-based composite films were synthesized by melt mixing, followed by compression molding, to study the influence of titanium oxide nanoparticles (TONPs) and/or multi-walled carbon nanotubes (CNTs) on the thermal properties of LDPE matrices. Fourier transform infrared (FTIR) spectroscopy confirmed the slight increase in the band intensities after TONP addition and a remarkable surge after the incorporation of CNTs. The value of crystallization temperature (*T*_c_) was not modified after incorporating TONPs, while an enhancement was observed after adding the hybrid fillers. The melting temperature (*T*_m_) was not changed after introducing the CNTs and CNT/TONP hybrid fillers. The percentage crystallinity (*X*_c_ %) was increased by 4% and 6%, after incorporating 1 wt % and 3 wt % CNTs, respectively. The TONP incorporation did not modify the *X*_c_ %. Moreover, thermal gravimetric analysis (TGA) thermograms confirmed the increased thermal stability after introducing CNTs and hybrid fillers compared to TONP incorporation.

## 1. Introduction

The materials manufactured from polyolefins, such as polyethylene (PE) and polypropylene (PP), have large commercial production due to their durability, light weight, low cost, and excellent mechanical properties [1]. Fabricating thermally stable polymers is highly needed in modern society [2]. Polymer nanocomposites are potential materials for different applications because of their unique properties [3,4,5,6,7,8]. Low-density polyethylene (LDPE) is a common polymer applied in different uses including industrial and space applications [9,10,11,12,13]. LDPE is widely used in the space craft industry because of its interesting characteristics, including chemical resistance, high flexibility, light weight, low cost of processing, and excellent tensile and thermal stability properties [14,15,16]. When titanium oxide nanoparticles (TONPs) are incorporated into the polymer, a system for biomedical applications is formed for antibacterial activity and odor inhibition [17,18]. Zapata et al. [19] reported the effect of TO and TONP dispersion on the dynamic, mechanical, thermal, and catalytic behaviors of PE and linear LDPE (LLDPE) composites. Ethylene homopolymer and ethylene-*co*-1-octadecene copolymers with 0.063 mol/L (LLDPE-1) and 0.38 mol/L (LLDPE-2) were prepared using a metallocene catalyst to prepare in situ TO-based composites. It was realized that the dispersion of TO in PE galleries was better than that in LLDPE-2 chains. The thermal stability of the polymers was improved significantly after TONP incorporation. The decomposition temperature of the prepared composites was raised by 25 °C compared to the neat polymer. The mechanical results of TO/PE were different from that of TO/LLDPE-1 nanocomposites. Moreover, the tensile characteristics of TO/PE and TO/LLDPE-1 composites did not change compared to the neat polymer. The Young’s modulus and yield stress of LLDPE-2 galleries were improved after incorporating 3 wt % and 5 wt % TONPs.

Carbon nanotubes (CNTs) represent a common promising reinforcing filler for polymers, as they can enhance the thermal and mechanical properties of the pristine polymers [20,21,22]. The topology and the final properties of polymer/CNT composites are controlled by the nature of chemical interactions between the polymer galleries and CNTs [23]. The impact of multi-walled CNT loadings on the thermal characteristics of polypropylene (PP) composites was investigated by Zhou et al. [24]. The results showed that the decomposition temperatures were shifted to higher values, and the maximum mass loss rate declined roughly with increasing CNT weight fraction. Furthermore, the residues increased linearly with higher CNT contents. A 10% increase in tensile stiffness and a slight rise in tensile strength were observed in a multi-walled CNT/polystyrene (MWCNT/PS) composite system [25]. Recent reports mentioned the limited uses of natural fiber/polymer composites because of their low impact behavior [26]. Of these, Puttegowda et al. discussed the potential aerospace uses of synthetic polymer composites [27]. In addition, the application of polyhedral oligomeric silsesquioxanes (POSSs) for producing composites was developed extensively in recent years because POSSs exhibit the property of being molecules when compared with the other most commonly applied fillers [28]. In addition, polyvinyledene fluoride (PVDF) matrices exhibited promising characteristics when reinforced with CNTs and other fillers [29,30,31]. Our team worked on different types of hybrid composites [32,33,34]. Based on the abovementioned significance of these two fillers, this work pursues a means of comparative analysis of LDPE nanocomposite films with different types of additives. The influence of TONPs, CNTs, and TONP/CNT combinations on the morphological and thermal properties of LDPE matrices is discussed in this paper. This work aims to realize the effect of hybrid filler content on the thermal behavior of LDPE matrices.

## 2. Materials and Methods

### 2.1. Materials

LDPE used in the composite films was procured from Qatar Petrochemical Company (QAPCO). Titanium (IV) oxide anatase nanopowder (TONPs) (particle size of 15 nm, 232033) and multi-walled carbon nanotubes (CNTs; outer diameter × length: 6–9 nm × 5 µm; bulk density of 0.22 kg/m^3^; <95% carbon; 724769-25G) were obtained from Sigma-Aldrich (Saint Louis, MO, USA). All materials were used without any treatment.

### 2.2. Preparation of TONP/LDPE, CNT/LDPE, and TONP/CNT/LDPE Composite Films

**Figure 1:**
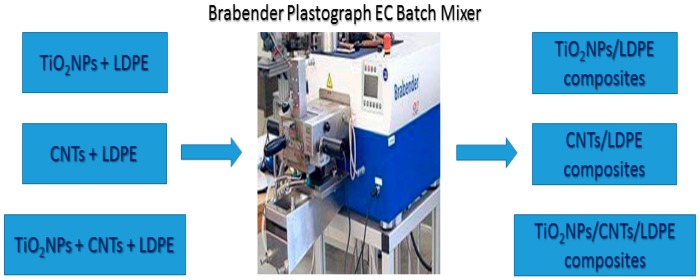
Illustrative diagram of mixing procedure.

LDPE-based composites were prepared using Brabender Plastograph EC batch mixer (Figure 1). The composites were blended with different contents of LDPE, TONPs, and/or CNTs to study the effect of filler content on the structural and thermal characteristics of LDPE matrices. The detailed composition of each sample and their designations are shown in Table 1. The mixing step was carried out at 180 °C and 50 rpm for a total duration of 10 min [35,36,37]. Then, the mixed composite lumps were pressed into sheets using a compression mounting press at 2 tons for 5 min at 180 °C. Finally, all prepared sheets were cooled to room temperature.

### 2.3. Film Characterization

#### 2.3.1. X-ray Diffraction (XRD)

The morphology of LDPE composites was investigated using XRD measurements. A MiniFlexII X-ray Diffractometer (Rigaku, Tokyo, Japan) was equipped at room temperature to perform XRD analysis of the neat LDPE and LDPE-based composite films. This technique was employed with a Cu-Kα source (*λ* = 1.5404 Å). It was performed at 50 kV and 20 mA [38,39,40]. The results were achieved in a 2*θ* range from 2° to 80°, with a 0.1° step size.

#### 2.3.2. Scanning Electron Microscopy (SEM)

A NOVA NANOSEM 450 (FEI^TM^, Brno, Czech Republic) was used to investigate the composite structure (voltage of 500 V to 30 kV). All as-prepared films were firstly plasma-etched to study the dispersion of the fillers into LDPE matrices. The samples with a fractured surface were produced by breaking the composites in liquid N_2_. All films were then coated by a thin film of gold in a sputtering chamber on the SEM holder.

#### 2.3.3. Fourier Transform Infrared Spectroscopy (FTIR)

A Frontier FTIR spectrometer (Perkin Elmer, Shelton, CT, USA) was used to investigate the interfacial interactions between the LDPE galleries and the additives using FTIR spectra. This spectrometer worked in attenuated total reflectance (ATR) mode. The spectra of the films were measured in the range of 4000–450 cm^−1^.

#### 2.3.4. Differential Scanning Calorimetry (DSC)

Differential scanning calorimetry was performed to determine the melting temperature (*T*_m_), crystallization temperature (*T*_c_), and percentage crystallinity (*X*_c_ %) using a Jade DSC (PerkinElmer, Shelton, CT, USA). Initially, 5 mg of all films were heated from 30 °C to 180 °C, before being cooled to 30 °C and heated again to 180 °C. This procedure is a typical one that is used in the literature to conduct DSC experiments [41]. The first scan is to erase the thermal hysteresis of the sample, while the cooling is to record the recrystallization, and the third step is to record the *T*_m_ value [41]. The heating and cooling rates were set at 10 °C/min. Nitrogen gas was purged during measurements. The enthalpy of the melting process value (∆*H*_f_) was determined in J/g from the measured *T*_m_ value. The *X*_c_ % was calculated as shown in Equation (1), where ∆*H*_o_ is the melting enthalpy of polyethylene of 100% crystallinity and is equal to 290 J/g [42].

(1) $$X_{c}\;\%=\frac{\left(∆H_{f}/W\%\right)}{∆H_{o}}\times 100.$$

#### 2.3.5. Thermal Gravimetric Analysis (TGA)

A Pyris 6 TGA (PerkinElmer, Shelton, CT, USA) was used to obtain TGA thermograms of weight loss as a function of temperature under nitrogen atmosphere for the neat LDPE and LDPE nanocomposites. Samples were heated from 30 °C to 600 °C with a heating rate of 10 °C/min.

## 3. Results and Discussion

### 3.1. Morphological and Dispersion Properties of LDPE-Based Nanocomposites

**Figure 2:**
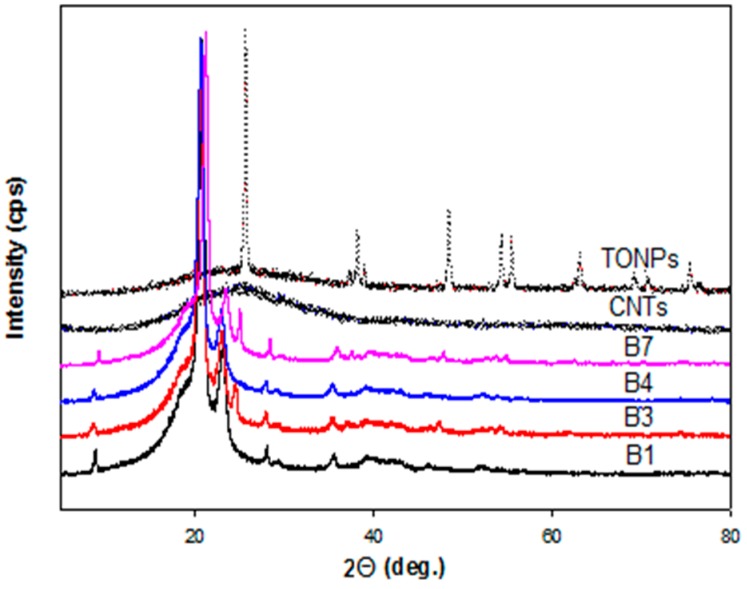
X-ray diffraction (XRD) patterns of TiO_2_ powder, carbon nanotubes (CNTs), pristine low-density polyethylene (LDPE), and the representative composite films.

Figure 2 represents the XRD spectra of LDPE and selected composites. The LDPE phase has two characteristic sharp peaks centered at 21.41° and 23.60°, corresponding to (110) and (200) planes, respectively [43]. The appearance of another characteristic peak at 25.05° in the composites having 2 wt % TONPs (B3 and B7) corresponds to the TiO_2_ anatase phase. Furthermore, CNTs exhibit a characteristic XRD peak centered at 23.4°, corresponding to the (002) plane [44]. This peak is not shown in the spectra of the composites having 1 wt % CNTs (B4 and B7). This disappearance confirms the good dispersion of CNTs within the LDPE network, and the same observation was reported by Yurdakul et al. [45].

**Figure 3:**
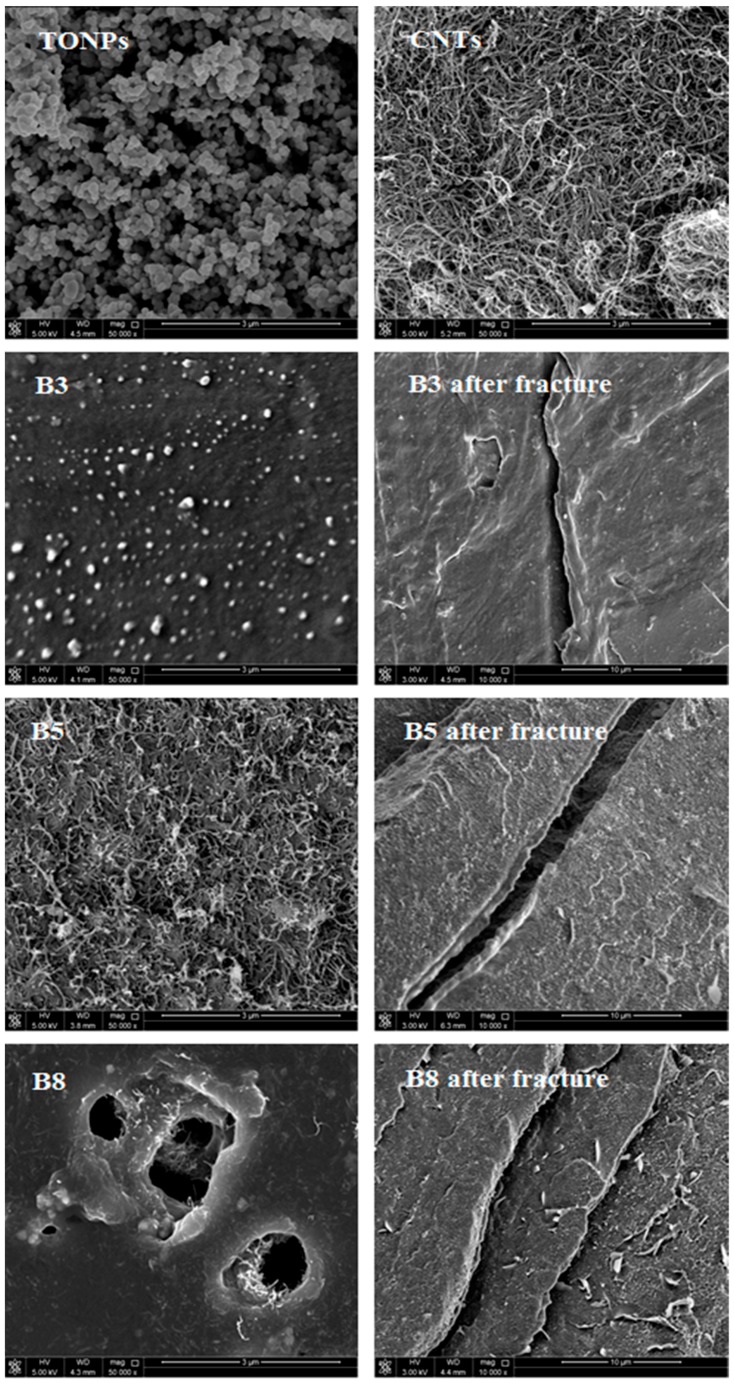
N-SEM images of titanium oxide nanoparticle (TONP) powder, CNTs, and the representative nanocomposites.

Surface topology of the additives and their dispersion into the polymer galleries was studied by Nano-SEM (N-SEM) and the representative images are demonstrated in Figure 3. This figure also compares the typical SEM micrographs of the composite films and the fractured surface. The images of the pure additives realized a spherical morphology of TONPs, while CNTs were tubular clusters and existed in entangled agglomerates. A micrograph of the sample containing 2 wt % TONPs (B3) confirmed that TONPs floated and dispersed well on the LDPE network, as shown in Figure 3. Floatation of the TONPs may be due to the poor compatibility between TONPs and LDPE chains. The dispersion of CNTs into the LDPE matrix was also poor, and entangled agglomerates of CNTs can be seen from the micrograph of the film containing 3 wt % CNTs (B5). The surface morphology of the composite films containing 2 wt % TONPs and 3 wt % CNTs (B8) illustrate that there are some pores on the surface owing to plasma-etching. Furthermore, CNTs and TONPs were well dispersed and embedded throughout LDPE galleries. In such systems, the interactions were maximized; this can lead to remarkable changes in the thermal properties, as discussed in subsequent sections.

**Figure 4:**
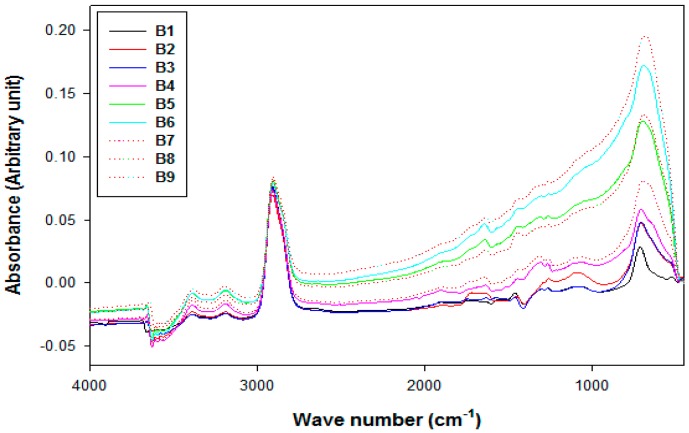
Fourier transform infrared (FTIR) spectra of LDPE and its composite films.

The fractured surface after breaking in liquid nitrogen and adding 2 wt % TONPs had fewer tracks compared to the 3 wt % CNT-filled films and was almost the same after simultaneous incorporation of 2 wt % TONPs and 3 wt % CNTs. This observation may be due to strong TONP–LDPE interfacial adhesion forces. The chemical interactions of fillers with the LDPE matrices were studied using FTIR spectroscopy, as shown in Figure 4. The pristine LDPE film demonstrates unique absorbance peaks at 2913, 2853, 1472, 1463, 729, and 719 cm^−1^ which correspond to CH_2_ bending and stretching vibrations of the LDPE network [46]. The chemical composition of LDPE did not change after the addition of TONPs and/or CNTs. Furthermore, the band intensities increased slightly after adding 1 wt % and 2 wt % TONPs, while the incorporation of CNTs led to a significant rise in the band intensities due to enhanced crystallinity (Figure 4), which is discussed in the subsequent sections [47]. The 719 cm^−1^ peak shifted to lower wave number if TONPs or/and CNTs were added. This shift may be attributed to the intermolecular interactions and forces between fillers and polymer chains [48,49]. The interfacial adhesion forces and intermolecular interactions between the fillers and LDPE matrices were confirmed upon correlating FTIR and SEM measurements.

### 3.2. Thermal Behavior of LDPE-Based Nanocomposites

DSC thermograms were used to investigate the influence of TONP and/or CNT incorporation on *T*_m_ and *T*_c_ values of LDPE-based composites. Table 2 reports the DSC values of neat LDPE and LDPE-based composite films. From Table 2, it can be observed that the neat LDPE exhibited a *T*_c_ of 93 °C. The incorporated TONPs did not change the value of *T*_c_ significantly. In contrast, a rise in *T*_c_ value of 3 °C was observed after incorporating 1 wt % CNTs. This increase can be ascribed to their role as a nucleating agent which offers nucleation positions on nanotube sidewalls for the LDPE matrix [50]. The *T*_c_ increased by 4 °C for 3 wt % CNTs, and then reduced again to a *T*_c_ of 96 °C for 5 wt % CNTs. Adding the hybrid additives increased the *T*_c_ and the ability of crystals to be formed at higher temperatures. The hybrid additive encouraged the growth and formation of crystallites in the polymer, as reported elsewhere [51,52]. The *T*_m_ after adding 1 wt % and 2 wt % TONPs increased by about 1 °C. It was not changed or modified after adding the CNTs. The same constant *T*_m_ was noticed for the hybrid additives. The *X*_c_ % calculated from DSC curves of the LDPE network increased after adding 1 wt % and 3 wt % CNTs by 4% and 6%, respectively. For the addition of 5 wt %, *X*_c_ % increased by 4%. The TONP addition did not change the *X*_c_ % much. The hybrid additives in B7 had a high *X*_c_ % value of 34%. The hybrid additives increased the packing of chains, and encouraged crystallinity.

**Figure 5:**
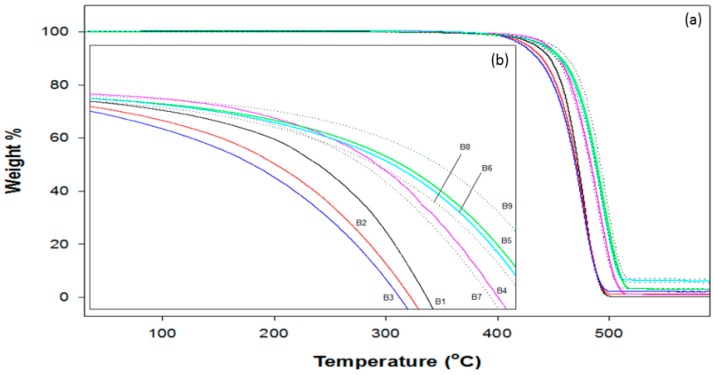
(**a**) Thermal gravimetric analysis (TGA) curves of pristine LDPE and its composite films. (**b**) Expanded degradation curves.

TGA thermograms of neat LDPE and LDPE composites are shown in Figure 5. The pristine LDPE had single-stage degradation with 80% weight loss at 458 °C due to the C–C bond cleavage without leaving any residue [53,54,55,56]. In general, major weight losses were noted in the range of 400–550 °C, which corresponds to polymer degradation. Evidently, the thermal stability of TONP/LDPE systems was reduced compared to the pristine LDPE film. The TGA results of CNT/LDPE composites were quite in contrast to the corresponding ones reported earlier by Yang et al. [57]. The presence of hybrid additives increased the thermal stability of the composites, and this behavior was ascribed to the better dispersion of the fillers in the matrix [34,58]. This influence may be ascribed to the incorporated CNTs effectively preventing the diffusion of volatile decomposition products and the creation of stable free radicals [59,60]. After 550 °C, all TGA thermograms became flat, and mainly the residue remained. The TGA curves, highlighted in Figure 5, clearly reveal that the initial decomposition temperature (*T*_i_) values attained for all CNTs and hybrid composites were higher than that of LDPE, thus emphasizing that the dispersion of CNTs in the polymer network does enhance the thermal stability of these composites. In contrast, the *T*_i_ values of TONP/LDPE systems were lower than that of LDPE, suggesting that the incorporation of TONPs does not offer thermal stability to these systems.

It can be revealed that the intermolecular interactions and interfacial adhesion forces between TONPs and/or CNTs with LDPE matrices were confirmed from the XRD, SEM, FTIR, DSC, and TGA experiments discussed in this work, and how the fillers’ interactions enhanced the thermal properties of LDPE matrices by creating stable free radicals and preventing the diffusion of volatile decomposition products.

## 4. Conclusions

A successful method for enhancing the morphological and thermal characteristics of LDPE matrices was achieved by adding different contents of TONPs, CNTs, and TONPs/CNTs. The nanocomposites were processed by melt mixing followed by compression molding to prepare thin films. The added TONPs did not modify the value of *T*_c_. On the other hand, an increase in *T*_c_ value of 3 °C was noticed after adding 1 wt % CNTs. Introducing the hybrid fillers enhanced the *T*_c_ and the ability of crystals to be produced at higher temperatures. The *T*_m_ after adding 1 wt % and 2 wt % TONPs increased by 1 °C. It was not modified after incorporating the CNTs and CNT/TONP hybrid fillers. The *X*_c_ % was improved after adding 1 wt % and 3 wt % CNTs by 4% and 6%, respectively. The TONP incorporation did not modify the *X*_c_ %. Furthermore, TGA curves showed the improved thermal stability after adding CNTs and hybrid fillers as compared to TONP addition. It can be summarized that the intermolecular interactions between TONPs and/or CNTs with LDPE matrices were confirmed from the different experiments addressed in this work, and how CNTs’ interactions improved the thermal stability of LDPE matrices by preventing the diffusion of volatile decomposition products and creating stable free radicals.

## Figures and Tables

**Table 1:** Designation and formulation of the synthesized low-density polyethylene (LDPE)-based composite films. TONP—titanium oxide nanoparticle; CNT—carbon nanotube.

Sample Designation	wt % TONPs	wt % CNTs	wt % LDPE
B1	0	0	100
B2	1	0	99
B3	2	0	98
B4	0	1	99
B5	0	3	97
B6	0	5	95
B7	2	1	97
B8	2	3	95
B9	2	5	93

**Table 2:** *T*_m_, *T*_c_, ∆*H*_m_, and *X*_c_ % of the pristine LDPE and its composite films.

Sample Designation	$T_{m}$ (°C)	$T_{c}$ (°C)	∆*H*_f_ (J/g)	*X*_c_ %
B1	108	93	87	30
B2	110	94	88	30
B3	110	94	90	32
B4	108	96	99	34
B5	109	97	102	36
B6	109	96	95	34
B7	109	96	94	34
B8	108	96	81	30
B9	108	96	82	30
